# A comparison of techniques to address the frequency of *Helicobacter pylori* positive dyspeptic patient

**DOI:** 10.1186/s13104-018-3897-1

**Published:** 2018-11-03

**Authors:** Priyatam Khadka, Ganesh Chapagain, Govinda Maharjan, Prakash Paudyal

**Affiliations:** 1 Trichandra Multiple Campus, Tribhuvan University, Ghantaghar, Kathmandu, Nepal; 2 Nepal National Hospital, Kathmandu, Nepal

**Keywords:** Diagnostic test, Functional dyspepsia, *Helicobacter pylori*, Low-income countries

## Abstract

**Objectives:**

The study was aimed to compare the diagnostic techniques, for the detection of *Helicobacter pylori* infection, available in low-income countries, where molecular testing is not available or inaccessible to anyone.

**Results:**

Of total enrolled patient, with the mean age of 41.4 ± 13.33 years, 24 (14 female; 10 male) were diagnosed to have been infected. The diagnosis was established based upon the gold standard test [either two of three tests: Rapid Urease Test (RUT), culture and histological examinations]. Of clinical presentation, the epigastric pain (75%) was the commonest; nevertheless, the endoscopic findings had shown an equivocal specificity since the larger percentile (58.3%) reported as normal findings, in a presumed dyspepsia. Based on the premise—with calculated sensitivity, specificity, and predictive values; the accuracy order observed as histology > RUT > serology > stool antigen test, in *H. pylori* detection from the clinical samples. The accuracy order of the diagnostic test may vary depending upon the laboratory settings and study population. Therefore, in reference to low-income countries, the clinicians must resort any available positive test so that the supporting positive rudiments would be an ancillary in augmenting the diagnostic accuracy.

## Introduction

*Helicobacter pylori*, a definite biological carcinogen as defined by WHO 1994, is of major concern today; because of its causal gastrointestinal diseases including gastritis, duodenal and gastric ulcers, non-ulcer dyspepsia, gastric adenocarcinoma, and lymphoma [1, 2]. *H. pylori* is one of the most successful human pathogen, since many chronic viruses infect close to 100% of world population [3]. Meanwhile, relating the clinical presentations, nearly 5% of functional dyspepsia may be attributable to the pathogen [4].

Dyspepsia is an extremely common disorder in an otherwise healthy individual comprising an assorted range of chronic upper abdominal symptoms which resembles the different gastrointestinal disorders: reflux disease, irritable bowel disease, gallbladder, and pancreatic dysfunction, and celiac disease [5, 6].

The chronic or recurring epigastric pain or discomfort centered in the upper abdomen for which no associated organic disease can be determined, classically described as the functional dyspepsia [6]. The clinical diagnosis, in general, presumed as functional dyspepsia or investigated dyspepsia when other possible gastrointestinal diseases along with a macroscopic lesion observed on upper endoscopy were excluded [6]. The conditions appear more frequently in the clinical practices i.e. of the global prevalence 7–45% in an uninvestigated functional dyspepsia [7]. Hence, an early and precise diagnosis of the etiology, in a presumed dyspeptic patient is crucial for therapeutic interventions. At this time, a myriad of invasive and non-invasive tests are implicated in diagnosis; however, applicability testing, clinical circumstances and cost-effectiveness of the test strategy are obligatory—particularly in low-income countries where molecular testing is not available or unreached to anyone.

## Main text

### Materials and methods

#### Patient selection

Between, January and June 2018, the consecutive outpatients receiving diagnostic endoscopy at the Department of Gastroenterology, Nepal National Hospital for unexplained dyspepsia were enrolled in our study.

#### Inclusion criteria

The patients, equal number of male and female, visiting Nepal National Hospital with uninvestigated symptoms of dyspepsia with gastrointestinal symptoms: upper abdominal pain or discomfort, bloating, nausea and vomiting, or early satiety, were included in the study.

#### Exclusion criteria

The patient who underwent partial or complete gastrectomy or major abdominal surgery, those with a history of peptic ulcer organic and metabolic diseases, use of steroids or non-steroidal anti-inflammatory drugs, or those with *H. pylori* eradication therapy or under any antimicrobial therapy within past 4 weeks were excluded from the study.

The research protocol was approved by the local ethical committee of the Nepal National Hospital. A written informed consent was obtained from every patient prior endoscopy, and sample collection.

#### Endoscopy and biopsy sampling

After an overnight fast, the endoscopic procedures were performed by the Gastroenterologist. Four gastric biopsies (two from the antrum and two from the corpus) were taken from each patient. Of total four samples, two of them—one from corpus from antrum—were fixed in 10% formalin in different vials and were sent for histopathological examination. Similarly, two of them—one from corpus from antrum were sent to the microbiology laboratory of the Nepal National Hospital for culture and urease test following aseptic measures.

#### Microbiological culture and Rapid Urease Test (RUT)

The RUT was performed from the non-commercial validated test. The reagent preparation was done as per standard microbiological methods recommended by American Society of Microbiology (ASM). In brief: 1 mL distilled water + 1 drop of 1% phenol red + 100 mg urea (Hi media, India), prepared just before endoscopy. The biopsy samples were stabbed, of two gastric biopsy specimens, the biopsy from antrum was placed in 1 mL urease broth for RUT; while biopsy from corpus was placed in Stuart’s transport medium for a microbiological culture.

Within a few minutes, the samples were processed, with grinding and streaking aseptically, with recommended methods. In brief, for microbial culture, the specimen was inoculated in Brain Heart Infusion (BHI) agar supplemented with: sheep blood (10%), amphotericin (5 mg/L), cefsulodin (5 mg/L), trimethoprim lactate (5 mg/L), and vancomycin (10 mg/L); incubated under micro-aerophilic condition (5% O_2_, 10% CO_2_, and 85% N_2_) at 37 °C for 7 days. As for the presumptive bacterial growth monitoring, the minute colonies were stained on gram stain and observed for the gram-negative curved bacilli. Further, identification of growth of the bacteria was done following standard microbiological methods recommended by American Society of Microbiology [8, 9].

For RUT, the specimen was inoculated in 1 mL prepared urease broth for 2 h at room temperature and observed for pink color on the positive test.

#### Histological examination

For histological examination, the specimens were fixed with 10% formalin (not more than a week), embedded in paraffin, stained with Giemsa then observed microscopically for spiral bacteria.

#### Serology

On the same day as biopsies from patients undergoing endoscopy, 1 mL of sera was collected, aliquoted, and stored at − 20 °C until used. The serological assay for detection of IgG antibodies against *H. pylori* was performed by a commercial *H. pylori* IgG ELISA kit (IBL, Hamburg, Germany) according to the manufacturer’s instructions. The cutoff value was assessed as per the manufacturer’s manual for the positive or negative test. As anti-*H. pylori* immunoglobulin (Ig) G titers were > 12 U/mL, the results were interpreted as positive, negative if they were < 8 U/mL, and equivocal if they were between 8 and 12 U/mL.

#### *Helicobacter pylori* stool antigen test (HpSAg)

The stool antigen test for *H. pylori* was performed, as per the manufacturer instructions, in ImmunoCard STAT! HpSAg-test kit (later ImmunoCard) Meridian Bioscience Europe—based on monoclonal *H. pylori* antibodies and a lateral flow chromatography technique. In brief, a small portion (approx. 5–6 mm diameter) of stool specimen was transferred in sample diluents vial and mixed thoroughly. The diluted stool sample was dispensed to the sample well of the test cassette; was observed for pink-red line in the reading window on positive test after incubation of 5 min at room temperature.

The sensitivities and specificities of the tests were calculated assuming the results (from culture, RUT, histopathological examinations) as gold standard in diagnosis of *H. pylori* infection [10]. If 2 of 3 tests were positive, the patients were considered to be infected with *H. pylori* (true positive); as if all three tests found negative were considered of not having *H. pylori* infection (true negative).

### Results

#### Patient demographics

During the study period, out of 90 patients—with an uninvestigated symptom of dyspepsia and/or having gastrointestinal symptoms—enrolled as study subjects. As urea breathe test was not performed in our study (culture, RUT, histopathological examinations) were considered as gold standard for determination of sensitivities and specificities of the tests. Relying upon the stated criteria—at least two of three tests are positive; 24 (26.6%) of the patients were diagnosed as the positive cases. Of reported positive cases, 14 (58.33%) were female and 10 (41.7%) male. The age ranged from 20 to 80 years, with mean age 41.4 ± 13.33 years.

#### Clinical presentation and endoscopic findings

Relating clinical presentations to the infection acquisition; the commonest, being the epigastric pain (75%). However, dyspeptic symptoms (45.80%), nausea and vomiting (29%), alteration of bowel habits (16.6%), and loss of appetite (12.5%), were found as a non-specific clinical presentation in *H. pylori*-positive patients, as shown in Table 1(i).

**Table 1:** The clinical presentation and endoscopic findings in the presumed dyspeptic patient

Clinical presentation	No. of case (%)	*H. pylori* positive case (%)
(i): Clinical presentation in presumed *H. pylori* positive dyspeptic patient		
Alternation of bowel habit	24 (26.6)	4 (16.6)
Abdominal pain	81 (90)	18 (75)
Dyspeptic symptoms	66 (73.3)	11 (45.8)
Nausea and vomiting	69 (76.6)	7 (29)
Loss of appetite	41 (45.5)	3 (12.5)

Endoscopic findings	No. of case (%)	*H. pylori* positive (%)
(ii): Endoscopic findings in *H. pylori* positive case		
Normal	12 (13.3)	7 (58.33)
Gastritis	49 (54.4)	8 (16.32)
Duodenitis	19 (21.1)	5 (26.3)
Ulcers	6 (6.6)	3 (50)
Malignancy	4 (4.4)	1 (25)

Total enrolled case = 90; total *H. pylori* positive case = 24

The endoscopic findings had shown an equivocal specificity; since the larger percentile was reported as normal findings (58.3%). The patients with ulcers (50%), duodenitis (26.3%), malignancies (25%), and gastritis (16%); however, found positive for the infection, as shown in Table 1(ii).

#### Comparative diagnostic methods

The biopsy based and serological assay based diagnostic test were analyzed for their sensitivities, specificities, predictive values, and accuracy in relation to the presumed gold standard (culture, RUT, histopathological examinations i.e. at least two of three test). The comparisons of different methods in diagnosis of *H. pylori* infection are shown in Table 2.

**Table 2:** Comparison of different methods in the diagnosis of *H. pylori* infection

Test	Gold standard		Sensitivity	Specificity	Positive predictive value (%)	Negative predictive value (%)	Accuracy (%)
	Positive	Negative					
Histology	21	69	80.9	94.2	80.90	94	91.10
Rapid Urease Test (RUT)	34	56	82.3	87.5	80.00	86	85.50
Serology (IgG ELISA)	66	24	89.3	54.1	84.20	65.00	80
Stool antigen test (HpSAg)	39	51	53.8	88.2	78	71	73.30

### Discussion

Approximately, a half of the world population found traumatized with the *H. pylori* infection; regrettably, 90% of the estimated prevalence in developing countries [11–13]. It has been observed, in reference with a myriad of epidemiological studies, the significant differences in prevalence across the world; which relies particularly on socioeconomic conditions like overcrowding, poor sanitation, hygiene and behavior traits of the patient [11, 14–16]. In an aforementioned epidemiological study, the prevalence of *H. pylori* in dyspeptic patients reported 38.4%, based on the PCR findings, in Nepal [17]. However, the accessibility of molecular testing, in most of the laboratory settings—principally in low-income countries, is beyond the reach of anyone. Hence an elucidation of all possible direct and indirect tests, with their calculated sensitivities, specificities, predictive values, and accuracies is necessary while opting as the diagnostic test.

An equal number of the male and female, although, enrolled in the study; larger number of the female having *H. pylori* infection was observed. The finding was consistent with the study of Miftahussurur et al. who found a similar female preponderance in dyspeptic Nepalese population [17].

Referring to our findings, on equating clinical presentation to the positive case: 75% had epigastric pain, 45.8% had dyspeptic symptoms, alternation of bowel (16.6%). El-Omer et al., however, in their study has shown: 80–90% of the dyspeptic patients have associated symptoms of epigastric pain, anorexia, nausea, vomiting, early satiety and regurgitation [18]. In contrast, Srinavas et al. reported the associated symptoms: epigastric pain (28%), nausea (0.1%), vomiting (23.2%), early satiety (0.18%), in the presumed *H. pylori*-infected patient [19].

Of total case enrolled, 12 of them had normal endoscopic findings; however, 7 (58.3%) found positive of harboring the infection. The percentile/findings were in line with the study by Okello et al. where he reported 51% patient with negative endoscopic findings in *H. pylori* positive cases [20]. Besides, among patients with positive endoscopic findings, inflammatory lesions like ulcers (50%) and duodenitis (26.3%) observed more frequently.

Among the diagnostic test implicated in our study, the histological examination reveals the higher accuracy (91.10%) with notable sensitivity (80.9%) and specificity (94.2%); the stool antigen test, nevertheless, shows lower accuracy (73.3%) with limited sensitivity (53.8%) and specificity (88.2%). In an epidemiological study, Miftahussurur et al. reported as the overall accuracy (97.3%), sensitivity (92.7%), specificity (100%) for histology; the findings are accordance with that our premises [17]. Similarly, Khalifehgholi et al. revealed, the accuracy, sensitivity, specificity for the histological test was 86.8%, 95.6%, 77.8% respectively. However, the stool antigen test compared to ours, Khalifehgholi et al. reported of higher accuracy (80.2%), sensitivity (73.9%), specificity (86.7%), in positive *H. pylori* cases [10].

Similarly, RUT reveals the accuracy (85.5%) with noteworthy sensitivity (82.3%) and specificity (87.52%); IgG serology (ELISA), nonetheless, shows the (80%) accuracy with limited sensitivity (89.3%) and specificity (54.1%). Nevertheless, in a similar type of study, Khalifehgholi et al. reported the higher accuracy, sensitivity, specificity: 97.8%, 95.6%, 100% respectively for RUT; nevertheless, for IgG serology the (73.6%) accuracy, sensitivity (91.3%) and specificity (55.6%) was elucidated [10].

Based upon the premise, the accuracy order of the diagnostic methods observed as histology > RUT > serology > stool antigen test. The order, however, had found variable in different cohort studies; possibly due to the pathogen variant and immune response to the infection of different study population [10].

Currently, urea breath test with improved infrared spectrometers have shown an extra advantage of low cost with augmented sensitivities and specificities. The test, nonetheless, is more suitable for the evaluation of *H. pylori* infection eradication or in follow-up after a subsequent antimicrobial therapy [21]. The sensitivity of the test was assumed quite well on post-therapy, however; the test was not included in the study since the study was not aimed at post-therapy patient [21, 22].

Moreover, in low resource settings, the RUT (as an invasive test) and stool antigen (as a non-invasive test) may have an important role in clinical management of the infection. However, owing to, the low validity in predicting *H. pylori* infection, the stool antigen testing could be the crucial diagnostic test in follow up patient—like urea breath test. Ultimately, which in-turn decreases the number of patients requiring invasive tests, and cost as well [23]. Likewise, for a rapid and presumptive diagnosis, RUT could be another choice for the clinicians due to its comparable sensitivities to gold standard test, in detection of the pathogen—particularly low-income countries where molecular testing may not be assessable to everyone.

### Conclusions

In limelight, due to a protean clinical presentation and non-specific endoscopic findings; the dyspeptic syndrome is of pivotal concern for the clinicians. The accuracy order of the diagnostic tests may vary. Therefore, in reference to low-income countries, it is obligatory to resort any available positive test so that the supporting positive rudiments would be an auxiliary in augmenting the diagnostic precision.

### Limitation of the study

The risk factor assessments, assessments of molecular methods in diagnosis, and follow-up treatment outcomes could have done; however, was not possible, owing to lack of resources and funding, in our setting.

## Abbreviations

ELISA: enzyme-linked immune sorbent assay
FD: functional dyspepsia
HpSAg: *Helicobacter pylori* stool antigen
*H. pylori*: *Helicobacter pylori*
PCR: polymerase chain reaction
RUT: Rapid Urease Test
